# Optimization of Sunflower Oil Transesterification Process Using Sodium Methoxide

**DOI:** 10.1100/2012/475027

**Published:** 2012-04-19

**Authors:** Sara KoohiKamali, Chin Ping Tan, Tau Chuan Ling

**Affiliations:** ^1^Department of Process and Food Engineering, Faculty of Engineering, Universiti Putra Malaysia, 43400 Serdang, Selangor, Malaysia; ^2^Department of Food Science & Technology, Islamic Azad University, Shahr e Qods Branch, Tehran, Iran; ^3^Department of Food Technology, Faculty of Food Science and Technology, Universiti Putra Malaysia, 43400 Serdang, Selangor, Malaysia; ^4^Institute of Biological Sciences, Faculty of Science, University of Malaya, 50603 Kuala Lumpur, Malaysia

## Abstract

In this study, the methanolysis process of sunflower oil was investigated to get high methyl esters (biodiesel) content using sodium methoxide. To reach to the best process conditions, central composite design (CCD) through response surface methodology (RSM) was employed. The optimal conditions predicted were the reaction time of 60 min, an excess stoichiometric amount of alcohol to oil ratio of 25%w/w and the catalyst content of 0.5%w/w, which lead to the highest methyl ester content (100%w/w). The methyl ester content of the mixture from gas chromatography analysis (GC) was compared to that of optimum point. Results, confirmed that there was no significant difference between the fatty acid methyl ester content of sunflower oil produced under the optimized condition and the experimental value (*P* ≥ 0.05). Furthermore, some fuel specifications of the resultant biodiesel were tested according to American standards for testing of materials (ASTM) methods. The outcome showed that the methyl ester mixture produced from the optimized condition met nearly most of the important biodiesel specifications recommended in ASTM D 6751 requirements. Thus, the sunflower oil methyl esters resulted from this study could be a suitable alternative for petrol diesels.

## 1. Introduction

In recent decades, the high global demand of energy along with the dramatic decrease in petroleum sources attracted world's attention to substitute any alternative sources for conventional petroleum fossil fuels. Biodiesels are fatty acid alkyl monoesters produced from reaction of low molecular alcohols (e.g., ethanol and methanol) with triacylglycerols. Since the important fuel properties of most of the alkyl monoesters are close to those of conventional diesel fuels, they can be considered as suitable alternatives for diesel fuels.

**Figure 1:**
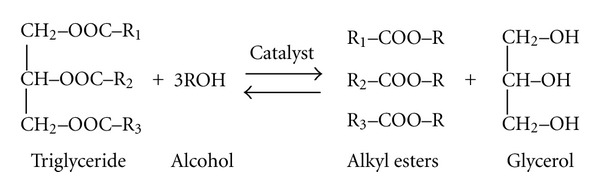
The general stoichiometric transesterification reaction diagram. Source: [1].

Furthermore, biodiesels are sources of energy, which are renewable, biodegradable, nontoxic, and environmental friendly. Transesterification (alcoholysis) is a method, in which triglyceride molecules (comprising 98% of vegetable oil's component) split up during the reaction of a monohydric alcohol with the glycerol part of triglyceride. Thus, the glycerol part would be replaced with the alkyl group of alcohol, which leads to a monoalkyl esters (biodiesels) formation (Figure 1) [1]. The main parameters recognized to have influences on transesterification reaction are the catalyst, alcohol amount, reaction time, temperature, free fatty acids (FFAs), and the presence of water in reactants [2].

Transesterification can be accelerated by either acidic or basic catalysts. The base-catalyzed reaction is preferred caused by being faster and less corrosive. In addition, the type of catalyst is reported to affect the phase separation (alkyl esters from glycerol). In this regards, strong bases such as potassium hydroxide (KOH), sodium hydroxide (NaOH) and sodium methoxide (CH_3_ONa) are usually used in the transesterification process. However, sodium methoxide reported to be the most active basic catalyst which induced the good phase separation [3]. Besides, Bacovsky et al. (2007) indicated that by using sodium methoxide, no more water would form, and later, the soap formation would be avoided [4]. 

From the stoichiometric view point and theoretically, in transesterification reaction, one mole of triglyceride requires three moles of alcohol (3 : 1 molar ratio of alcohol to oil) to form three moles of alkyl ester (Figure 1). However, in the factual reaction, an excess of alcohol is needed to shift the equilibrium to the right and raise the yield of the alkyl esters' production (Figure 1) [5]. This issue was, however, attributed to the volatile nature of the alcohol [6]. Moreover, the type of alcohol was shown to have great effect not only on reaction kinetics but also on fuel characteristics of the resultant product. Based on the literatures, the yield of biodiesel produced using methanol was higher than ethanolysis [7–10]. This phenomenon was attributed to the formation of stable emulsions between glycerol and alkyl monoesters during the ethanolysis [8, 11]. Thus, methanol was considered as the preferred alcohol of the alcoholysis and because of its low price, ease of reaction with triglycerides, and the immediate dissolution of the catalyst than other alcohols [1]. Also, the alcohol recommended being of anhydrous, since, otherwise the transesterification would be replaced by hydrolysis. During the hydrolysis, the FFAs would be formed rather than alkyl monoesters, and then the transesterification yield and phase separation would be declined [6, 9, 12]. Correspondingly, the free fatty acids (FFAs) present in feed stock were shown to have negative effect on transesterification regarding to reaction with basic catalyst and formation of soaps. In which, the phase separation stage will be more difficult and costly due to emulsification of soap with water. Therefore, in case of high FFAs content (more than 0.5%) of the feed stock oil, a pretreatment was highly recommended before transesterification [1, 7, 9].

Based on the previous research results, the ambient temperature was shown to be enough for transesterification reaction, while at higher temperatures, the maximum conversion time would be decreased [3]. In this study, the reaction temperature was fixed at 60°C, which was bellow the boiling point of methanol and thus the need for using a pressure vessel was excluded. Parameters considered influential on the transesterification process were being the reaction time, the mass ratio of alcohol to oil, and concentration of the catalyst.

The main aim of this project was to get to the highest methyl ester content from transesterification of sunflower oil than prior findings which can be suitable for petrol fuel replacement. In order to optimize the process, central composite design (CCD) and response surface methodology (RSM) were employed to predict the response for experimental design. Based on the CCD matrix, parameters varied within a defined range to reach an optimum condition for biodiesel production. Thereafter, some important fuel properties of the resultant biodiesel were assessed and compared with ASTM D 6751 standards.

## 2. Materials and Methods

### 2.1. Materials

Refined sunflower oil was purchased from Malaysian markets. Methanol (reagent grade) and n-heptane as a solvent for gas chromatographic (GC) analysis were purchased from Fisher Scientific (Pittsburgh, PA, USA). Pure methyl heptadecanoate (≥99.5%) as a reference internal standard and sodium methoxide as an alkaline catalyst were purchased from Sigma Chemical Co. (St. Louis, MO).

### 2.2. Production of Methyl Esters

This experiment was carried out using a laboratory scale reactor IKA (LR 2000V, Germany). The closed system consisted of a double jacket glass vessel equipped with a mechanical stirrer, water condenser, temperature regulator, sampling outlet, and an adjustable water bath providing the desired temperatures.

#### 2.2.1. Transesterification

The acid value of sunflower oil was measured [13]. Due to the negligible amount of free fatty acids (less than 0.5%), there was no need to perform the pretreatment before transesterification reaction [1, 7, 9]. According to the experimental design, 500 g of sunflower oil weighed, preheated nearly to the set temperature of 60°C. Then, the catalysts were dissolved in methanol and the fresh chemicals were added to the reactor entirely. A vigorous agitation (300 rpm) with a little splashing was applied to mix oil and alcohol phases [3, 14]. Based on the previous literatures, the methanolysis was started in a closed vessel and under a reflux condition for the suppression of any alcohol loss [1–3, 9, 14, 15].

#### 2.2.2. Phase Separation

Once transesterification finished at the specified time, the reaction progress was hindered through the addition of some drops of acidic water to the mixture [3]. Then, the mixture was transferred into a separatory funnel and was allowed to be settled for 1 h when separation of the product into two distinct layers was reached. The glycerol as a by-product was formed at the bottom layer and regarding to its higher density than that of fatty acid methyl esters (FAMEs) [1, 16].

#### 2.2.3. Neutralization

The methyl ester upper layer was then washed for four times using a warm mild acid solution (5% citric acid dissolved in distilled water at 50–60°C) followed by washing with a warm pure distilled water. This procedure repeated for another cycle [16]. The washing process, performed to decompose the soap formed during the transesterification reaction. Besides which, unreacted mono- di- and triglycerides, the residual alcohol and alkaline catalyst were also removed [1, 3, 9]. Moreover, through employing the warm (50–60°C) and mild acid solution, formation of immiscible ester/water emulsions and sedimentation of saturated fatty acids was avoided. Besides, due to the tendency of the esters to emulsion formation, agitation of the mixture during the washing process was kept to a minimum [3].

#### 2.2.4. Distillation

Later, the methyl ester fraction was distilled off using the rotary vacuum evaporator for 1 h at 80°C and under a moderate vacuum for elimination of residual water and methanol [6, 9]. The resulted fatty acid methyl ester rich mixture was then stored under the nitrogen at −18°C (due to oxidative instability of the product) before the gas chromatography analysis [17].

### 2.3. Design of Experiment and Analytical Methods

#### 2.3.1. Design of Experiment

Response surface methodology (RSM) was applied to design the matrix of experiments (Table 1) to investigate the effect of three main independent variables on methyl ester content (*y*, w/w%) of sunflower oil as the response. The factors chosen were the reaction time (*x*_1_, 60–180 min), the excess stoichiometric amount of methanol to oil (*x*_2_, 25–125 w/w%), and the catalyst concentration (*x*_3_, 0.1–0.9 w/w%).

The type of catalyst and alcohol, the agitation rate and reaction temperature were kept fixing during the experiments. Therefore, twenty transesterification trials were designed based upon central composite design (CCD) (Table 1). The designed matrix incorporated 3 independent variables with 5 levels for every factor. The system repeatability was determined through repeating the center point (6 times) (treatment nos. 1, 4, 7, 11, 14, and 20) (Table 1). Where, the sequence of experiments was randomized for the sake of reducing the effects of any uncontrolled factor on response [12, 18].

#### 2.3.2. Internal Standard Solution and Sample Preparation

The methyl heptadecanoate in heptane (10 g/mL) solution was prepared as an internal standard. Roughly, 250 mg of each transesterified sample (FAMEs) is accurately weighed and diluted with 5 mL of internal standard solution [19].

#### 2.3.3. Gas Chromatography Analysis (GC)

Sample analysis was performed by gas chromatography using GC (Agilent 7890, Agilent Inc., DE, USA) equipped with FID (flame ionization detector) and autosampler injector. The injection was done at split mode (100 : 1) through using a highly polar BPX70 capillary column and 30 m length. The column temperature program raised from 140°C to 180°C at 10°C/min then to 220°C at the rate of 2°C/min and holding for 1 min. The inlet and FID temperatures were 230°C and 250°C, respectively. During which, the high-purity nitrogen with the flow rate of 0.7 mL/min was purged. The methyl esters content of each sample (*Y*, w/w%) was calculated using the following formula as a mass fraction in percent (1) [20]:

(1) $$\;Y=\frac{\sum A-A_{\text{IS}}}{A_{\text{IS}}}\times \frac{C_{\text{IS}}\times V_{\text{IS}}}{M}\times 100\%.$$

∑*A* = total peak area, *A*_IS_ = internal standard (methyl heptadecanoate) peak area, *C*_IS_ = concentration of the internal standard solution (mg/mL), *V*_IS_ = volume of the internal standard solution (mL), *M* = mass of the sample (mg).

### 2.4. Statistical Analysis and Model Fitting

As for the experimental design, data analysis was also performed through RSM and by using Minitab v.14, statistical package (Minitab Inc., PA, USA). The objectives of the data analysis were (1) to specify regression coefficients and recognizing significant model terms, (2) to fit the regression model and determine the factors optimum levels which leading to a greatest response.

Since the response was the methyl ester content (*Y*, w/w%), an empirical regression model was employed for a better understanding of the correlations between the factors and response applying a second-degree polynomial equation (2) [18]:

(2) $$Y=\beta_{0}+\overset{n}{\underset{i=1}{\sum }}\beta_{i}x_{i}\;\overset{n}{\underset{i=1}{\sum }}\beta_{ii}x_{i}^{2}+\overset{n-1}{\underset{i=1}{\sum }}\;\overset{n}{\underset{j=i+1}{\sum }}\beta_{ij}x_{i}x_{j},$$

where *Y* represents the predicted response; *β*_0_ is the offset term; *β*_*i*_ is the linear coefficients; *β*_*ii*_ and *β*_*ij*_ are the interaction coefficients; *x*_*i*_ and *x*_*j*_ are the independent variables [18].

 Since there were three factors involved in this study, the mathematical relationship between factors and the response becomes (3):

(3) $$\begin{array}{cc} Y & =\beta_{0}+\beta_{1}x_{1}+\beta_{2}x_{2}+\beta_{3}x_{3}+\beta_{11}x_{1}^{2}+\beta_{22}x_{2}^{2}\\ & \;+\;\beta_{33}x_{3}^{2}+\beta_{12}x_{1}x_{2}+\beta_{13}x_{1}x_{3}+\beta_{23}x_{2}x_{3}. \end{array}$$

The factors with smaller *P* values (*P* < 0.05) and larger extent of *t* value have the higher corresponding coefficients and then significant effect on response compared to other factors. So, the significant factors (*P* < 0.05) should be remained in the model, and ones with nonsignificant statistical effect (*P* > 0.05) are omitted. Then the model was rebuilt with only influential factors (*P* ≤ 0.05)  [18, 21]. Where the linear terms of some independent variables are nonsignificant (*P* > 0.05), they were still retained in the model, in favor of significance of their interaction or quadratic terms (*P* < 0.05) [21]. So the final empirical model suggested by the software and under uncoded units was as bellows (4):

(4) $$Y=96.38-0.794x_{1}+0.733x_{3}+0.528x_{2}^{2}.$$

As for a good fit model, it was recommended that, *R* ^2^ should not be less than 0.80 [22]. Thus, the variation of the response evaluated by measurement of coefficient of determination (*R* ^2^) [23, 24]. The value of *R* ^2^ at fitted model (0.889) substantiated that 88.9% of the total variation in response could be attributed to the experimental factors.

### 2.5. Optimization and Validation

**Figure 2:**
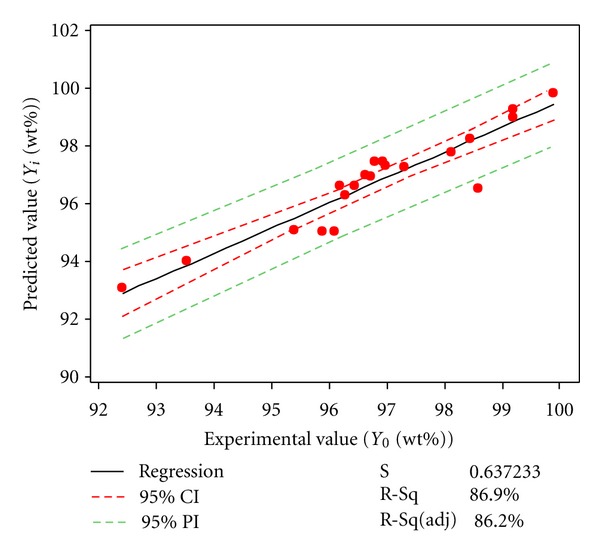
The fitted line plot indicating the correlation between the predicted and experimental (*Y*_0_) values of methyl esters (biodiesel) content. Fitted line plot for transesterification yield. Predicted value (*Y_i_*, %wt) = 11.99 + 0.8754. Experimental value (*Y_0_*, %wt).

The optimization process was carried out based on RSM procedures (Minitab software). The numerical optimization was conducted through response optimizer to get the optimal point, which resulted in the desired response. As the experimental and predicted values are shown in Table 2, the adequacy of the regression model was checked through comparison of the experimental and predicted data using the two-sample *t*-test (Figure 2) [25].

## 3. Results and Discussion 

The experimental data and predicted values are shown in Table 2. Although the methyl esters content from treatment numbers 5, 10, and 12 has shown to be in close proximity to 100%, the reaction time, amounts of alcohol, and catalyst applied were much higher than those of optimum point (Table 2). Thus, it is uneconomical and the operating conditions need to be optimized. 

 Regression coefficients, *F* ratio and *P* value of the model for linear, quadratic, and interaction effect of the terms with significant effects (*P* ≤ 0.05) were expressed in Table 3. 

 As shown in Table 3, the single effects of the reaction time (*x*_1_) and the catalyst concentration (*x*_3_) along with the quadratic effect of the excess stoichiometric amount of methanol to oil (*x*_2_∗*x*_2_) played important role on the variability response (*y*, w/w%) (*P* ≤ 0.05). The interaction terms of the variables have not shown to have any impressive effect on response (*P* ≥ 0.05). Consequently, except high-significant terms, the rests of others were dropped from the initial model to get to a fitted model. However, the main term of the excess stoichiometric amount of methanol to oil (*x*_2_) was still kept in the final model because of its quadratic term effect (*P* < 0.05). 

### 3.1. Effect of Reaction Time (*x*_1_)

**Figure 3:**
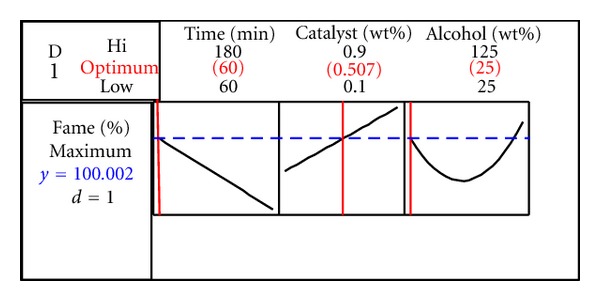
The response optimizer graph showing the optimum points.

The recorded results in Table 3 showed that the response was profoundly influenced by the reaction time (*P* < 0.05). The linear term of the reaction time, with the lowest *P* value and the highest *F*-ratio, was shown to be the most significant term (*P* < 0.05). According to the estimated coefficients from Table 3, the reaction time adversely affected the response. It is meaning that when the reaction time decreased, the greater amount of fatty acid methyl esters (FAMEs) would be produced. This result was in conformity with what Sanli and Canakci reported in 2008 [1]. Based on their outcome, the reaction time of 60 minutes was shown to be enough for getting the maximum methyl ester content. Since, the greater reaction time would result in reducing the conversion yield (Figure 3). This matter can be attributed to the ester hydrolysis after the formation [1]. Subsequently, the response surface optimizer suggested the reaction duration of 60 minutes in which the predicted response would be increased (Figure 3). 

### 3.2. Effect of Mass Ratio of Methanol to Oil (*x*_2_)

From Table 3, no significant effect of the linear term of alcohol (*x*_2_) on response was observed (*P* > 0.05). While, the quadratic term of alcohol (*x*_2_∗*x*_2_) significantly affected the response variable (*P* < 0.05). This result was in accordance with what Hameed et al. (2009) found [12].According to the response surface optimizer, the highest biodiesel content could be reached when the amount of alcohol is 25% more than that of stoichiometric one (about 3.75 : 1 molar ratio) (Figure 3). However, the excess amount of alcohol at the range of 25% to 75% displayed an intensive drop in response, caused by increasing the solubility of glycerol and the phase separation difficulties [9, 14, 26, 27]. 

Although the excess of alcohol at 75–125% resulted in increasing the response, but this predicted results were not too much higher than that of 25% excess of alcohol (Figure 3). Therefore, it would be reasonable to consider the ratio of methanol to oil at the optimal level, which is 25% and thus decrease the environmental contamination and extra costs of the process [1, 12, 14].

### 3.3. Effect of Catalyst Concentration (*x*_3_)

 As it could be seen, the linear term of the catalyst significantly affected the methylation yield (*P* < 0.05) (Table 3). In this regards, the corresponding estimated coefficient revealed a positive correlation between the linear term of catalyst concentration and the response. Through which it could be concluded that the rate of transesterification was accelerated by using the catalysts. Accordingly, the highest methyl ester's content was achieved when the catalyst concentration was 0.5% w/w of the oil. Interestingly, this amount of the catalyst lied in the range resulted from the previous studies [3, 5]. Since, the optimal amount of the catalyst displayed to be sufficient, increasing the catalyst amount not only does not enhance the response but also adds extra costs to transesterification process (Figure 3).

### 3.4. Validation of the Final Model

Through the *t*-test, no significant differences are observed between the experimental and predicted values (*P* ≥ 0.05). Moreover, the closeness of actual and predicted values in Figure 2 shows that the regression equation is adequate [21]. The GC analysis result from the empirically produced mixture under the optimized condition was compared to that of predicted from the software. The outcome, however, confirmed that there was no significant difference between the FAMEs produced under the experimental and predicted condition (*P* ≥ 0.05).

### 3.5. Fuel Characteristics 

The methyl esters mixture resulted from the sunflower oil methanoysis and under the optimized condition was evaluated based on ASTM (American Society for Testing Materials) standards [28–33]. In this regards, the results of quality assessment tests performed on the final product revealed that most of the fuel specifications comply with ASTM (American Society for Testing Materials) D 6751 requirements [34] (Table 4). 

## 4. Conclusion 

 The present research, however, suggested the optimum condition of 0.5% of NaOCH_3_, 60 min of time, 25% excess stoichiometric amount of methanol to oil, which is equal to 3.75 : 1 molar ratio and 60°C to achieve the optimum response in sunflower oil methnoysis. 

The response, which was the percentage of methyl esters in oil (100 w/w%), was higher than the recent results of Rashid et al. (2008) [9]. In comparison with Rashid et al. (2008) [9] results,the present study suggested a high yield, environmentally friendly approach, which reducedthe time of the process from 120 to 60 min, decreased the amount of alcohol from 6 : 1 to 3.75 : 1 and the catalyst from 1 to 0.5%. Since at transesterification, the catalyst is needed only in small amounts, the cost of the process is supposed to be relevant to the alcohol, which was shown to be sufficiently adjustable at optimal level (Figure 3). In addition, the biodiesel obtained by the optimal condition displayed to be of good quality and suitable for use in automotive engines and therefore can be incorporated to the renewable-energy sources.

## Figures and Tables

**Table 1:** The matrix of central composite design (CCD) resulted from response surface methodology design of experiment.

Treatment run	Blocks	Time of reaction (*x*_1_, min)	Excess stoichiometric amount of alcohol to oil (*x*_2_, w/w%)	Catalyst amount (*x*_3_, w/w%)
1^c^	2	120	75	0.5
2	2	150	50	0.3
3	2	90	100	0.3
4^c^	2	120	75	0.5
5	2	90	50	0.7
6	2	150	100	0.7
7^c^	1	120	75	0.5
8	1	150	50	0.7
9	1	150	100	0.3
10	1	90	50	0.3
11^c^	1	120	75	0.5
12	1	90	100	0.7
13	3	120	25	0.5
14^c^	3	120	75	0.5
15	3	60	75	0.5
16	3	120	125	0.5
17	3	120	75	0.9
18	3	180	75	0.5
19	3	120	75	0.1
20^c^	3	120	75	0.5
Opt. point*	—	60	25	0.5

^c^Center point.

^∗^The optimum point was resulted from the optimization procedure.

**Table 2:** Trial and predicted values for response variables of the reduced model.

Treatment run	Blocks	Methyl esters content^a^ (*Y*, w/w%)		
		Experimental value (*Y*_0_)	Predicted value (*Y*_*i*_)	*Y*_0_ − *Y*_*i*_^b^
1	2	96.46	96.64	−0.18
2	2	95.40	95.46	−0.06
3	2	97.32	97.61	−0.29
4	2	96.20	96.64	−0.44
5	2	99.20	99.21	−0.01
6	2	98.46	97.46	0.99
7	1	96.95	97.47	−0.52
8	1	98.14	98.08	0.05
9	1	97.00	96.49	0.51
10	1	99.20	98.23	0.96
11	1	96.80	97.47	−0.67
12	1	99.90	100.23	−0.33
13	3	96.64	96.99	−0.35
14	3	96.10	95.04	1.05
15	3	96.29	96.63	−0.34
16	3	96.99	97.31	−0.32
17	3	96.72	96.51	0.20
18	3	92.40	93.45	−1.05
19	3	93.54	93.57	−0.03
20	3	95.90	95.04	0.85
Opt. Point	—	99.70	100.04	−0.34

^a^No considerable differences (*P* > 0.05) between trial (*Y*_0_) and predicted values (*Y*_*i*_);

^b^ *Y*_0_ − *Y*_*i*_: residual.

**Table 3:** The regression coefficients (*R* ^2^), adjusted *R* ^2^, *F* ratio and *P* value of the final reduced model.

Parameter	Model term	Coefficient estimate	*F* ratio	*P* value
*β*_0_	Intercept	96.386	214122	0.000
*Linear*				
*β*_1_	*x*_1_	−0.79	24.51	0.000
*β*_2_	*x*_2_	0.080	0.25	0.625^a^
*β*_3_	*x*_3_	0.733	20.87	0.001
*Quadratic*				
*β*_11_	*x*_1_∗*x*_2_	—	—	0.532^a^
*β*_22_	*x*_2_∗*x*_2_	0.52	16.24	0.001
*β*_33_	*x*_3_∗*x*_3_	—	—	0.446^a^
*Interaction*				
*β*_12_	*x*_1_∗*x*_2_	—	—	0.175^a^
*β*_13_	*x*_1_∗*x*_3_	—	—	0.459^a^
*β*_23_	*x*_2_∗*x*_3_	—	—	0.550^a^
*R* ^2^	—	0.88	—	—
*R* ^2^ (adj)	—	0.83	—	—
Regression (*F*-ratio; *P* value)	—	—	239.32	0.000

^a^Nonsignificant effect (*P* > 0.05).

**Table 4:** Comparison of the fuel properties of the biodiesel resulted from the optimal condition, with ASTM D 6751 standard.

Fuel properties	Unit	Value	Standard limits	Standard methods
Specific gravity at 15°C	—	0.87	0.86–0.90	ASTM D 287
Kinematic viscosity at 40°C	m^2^/s	4.1 × 10^−6^	3.5 × 10^−6^ − 5 × 10^−6^	ASTM D 445
Flash point	°C	150	≥100	ASTM D 93
Pour point	°C	0	—	ASTM D 97
Ash content	w/w%	0.008	≤ 0.01	ASTM D 482
Cloud point	°C	2	−1	ASTM D 2500

ASTM; American Standards for Testing of Materials [28–34].

## References

[1] Sanli H, Canakci M (2008). Effects of different alcohol and catalyst usage on biodiesel production from different vegetable oils. *Energy and Fuels*.

[2] Ghadge SV, Raheman H (2006). Process optimization for biodiesel production from mahua (*Madhuca indica*) oil using response surface methodology. *Bioresource Technology*.

[3] Korus RA, Hoffman DS, Bam N, Peterson CL, Drown DC Transesterification process to manufacture ethyl ester of rape oil.

[4] Bacovsky D, Körbitz W, Mittelbach M, Wörgetter M (2007). Biodiesel production: technologies and european providers. *IEA task 39 report*.

[5] Dunford NT (1914). Biodiesel production techniques. *Food Technology Fact Sheets*.

[6] Klok R, Verveer HH

[7] Nye MJ, Williamson TW, Deshpande W (1983). Conversion of used frying oil to diesel fuel by transesterification: preliminary tests. *Journal of the American Oil Chemists’ Society*.

[8] Hossain ABMS, Boyce AN, Salleh A, Chandran S (2010). Impacts of alcohol type, ratio and stirring time on the biodiesel production from waste canola oil. *African Journal of Agricultural Research*.

[9] Rashid U, Anwar F, Moser BR, Ashraf S (2008). Production of sunflower oil methyl esters by optimized alkali-catalyzed methanolysis. *Biomass and Bioenergy*.

[10] Knothe G, Van Gerpen J, Krahl J (2005). *The Biodiesel Handbook*.

[11] Zhou W, Konar SK, Boocock DGB (2003). Ethyl esters from the single-phase base-catalyzed ethanolysis of vegetable oils. *Journal of the American Oil Chemists’ Society*.

[12] Hameed BH, Lai LF, Chin LH (2009). Production of biodiesel from palm oil (*Elaeis guineensis*) using heterogeneous catalyst: an optimized process. *Fuel Processing Technology*.

[13] American oil chemists’ society official and method. Reapproved Free fatty acid.

[14] Antolín G, Tinaut F, Briceo Y, Castao V, Pérez C, Ramírez A (2002). Optimisation of biodiesel production by sunflower oil transesterification. *Bioresource Technology*.

[15] Stamenković OS, Todorović ZB, Lazić ML, Veljković VB, Skala DU (2008). Kinetics of sunflower oil methanolysis at low temperatures. *Bioresource Technology*.

[16] Van Gerpen JH, Hammond EG, Yu L, Monyem A (1997). Determining the influence of contaminants on biodiesel properties. *SAE technical paper*.

[17] Leung YH, Liu RH (2000). *trans*-10,*cis*-12-conjugated linoleic acid isomer exhibits stronger oxyradical scavenging capacity than *cis*-9,*trans*-11-conjugated linoleic acid isomer. *Journal of Agricultural and Food Chemistry*.

[18] Montgomery D (2001). *Design and Analysis of Experiments*.

[19] Wang CX, McCurry J (2006). *Determining the Ester and Linolenic Acid Methyl Ester Content to Comply with EN14103*.

[20] Ruppel T, Huybrighs T (2008). *Fatty Acid Methyl Esters in B100 Biodiesel by Gas Chromatography (Modified EN14103)*.

[21] Mirhosseini H, Tan CP, Hamid NSA, Yusof S (2008). Optimization of the contents of Arabic gum, xanthan gum and orange oil affecting turbidity, average particle size, polydispersity index and density in orange beverage emulsion. *Food Hydrocolloids*.

[22] Joglekar AM, May AT (1987). Product excellence through design of experiments. *Cereal Foods World*.

[23] Weng WL, Liu YC, Lin CW (2001). Studies on the optimum models of the dairy product Kou Woan Lao using response surface methodology. *Asian-Australasian Journal of Animal Sciences*.

[24] Ahn JH, Kim YP, Lee YM, Seo EM, Lee KW, Kim HS (2008). Optimization of microencapsulation of seed oil by response surface methodology. *Food Chemistry*.

[25] Mirhosseini H, Tan CP, Taherian AR, Boo HC (2009). Modeling the physicochemical properties of orange beverage emulsion as function of main emulsion components using response surface methodology. *Carbohydrate Polymers*.

[26] Encinar JM, González JF, Rodríguez JJ, Tejedor A (2002). Biodiesel fuels from vegetable oils: transesterification of *Cynara cardunculus* L. Oils with ethanol. *Energy and Fuels*.

[27] Attanatho L, Magmee S, Jenvanitpanjakul P Factors affecting the synthesis of biodiesel from crude palm kernel oil.

[28] American Society for Testing and Materials. Reapproved Standard test method for API gravity of crude petroleum and petroleum products-Hydrometer method.

[29] American Society for Testing and Materials. Reapproved Standard test method for kinematic viscosity of transparent and opaque liquids. The calculation of dynamic viscosity.

[30] American Society for Testing and Materials. Reapproved Standard test methods for flash point by Pensky-Martens closed up tester.

[31] American Society for Testing and Materials. Reapproved Standard test method for pour point of petroleum products.

[32] American Society for Testing and Materials. Reapproved Standard test method for ash from petroleum products.

[33] American Society for Testing and Materials. Reapproved Standard test method for cloud point of petroleum products.

[34] American Society for Testing and Materials. Reapproved Standard specification for biodiesel fuel blend stock (B100) for middle distillate fuels.

